# The Amount of Keratins Matters for Stress Protection of the Colonic Epithelium

**DOI:** 10.1371/journal.pone.0127436

**Published:** 2015-05-22

**Authors:** M. Nadeem Asghar, Jonas S. G. Silvander, Terhi O. Helenius, Iris A. K. Lähdeniemi, Catharina Alam, Lina E. Fortelius, Rickard O. Holmsten, Diana M. Toivola

**Affiliations:** Cell Biology, Biosciences, Faculty of Science and Engineering, Åbo Akademi University, and Turku Center for Disease Modeling, Turku, Finland; RWTH Aachen, GERMANY

## Abstract

Keratins (K) are important for epithelial stress protection as evidenced by keratin mutations predisposing to human liver diseases and possibly inflammatory bowel diseases. A role for K8 in the colon is supported by the ulcerative colitis-phenotype with epithelial hyperproliferation and abnormal ion transport in K8-knockout (K8^−/−^) mice. The heterozygote knockout (K8^+/−^) colon appears normal but displays a partial ion transport-defect. Characterizing the colonic phenotype we show that K8^+/−^ colon expresses ~50% less keratins compared to K8 wild type (K8^+/+^) but *de novo* K7 expression is observed in the top-most cells of the K8^+/−^ and K8^−/−^ crypts. The K8^+/−^ colonic crypts are significantly longer due to increased epithelial hyperproliferation, but display no defects in apoptosis or inflammation in contrast to K8^−/−^. When exposed to colitis using the dextran sulphate sodium-model, K8^+/−^ mice showed higher disease sensitivity and delayed recovery compared to K8^+/+^ littermates. Therefore, the K8^+/−^ mild colonic phenotype correlates with decreased keratin levels and increased sensitivity to experimental colitis, suggesting that a sufficient amount of keratin is needed for efficient stress protection in the colonic epithelia.

## Introduction

Keratins (K) are intermediate filament (IF) proteins expressed in epithelial cells. In the simple-type epithelial of the small intestine and colon, type II keratins (K7 and K8) form filaments with type I keratins (K18, K19, K20, K23) as obligate heteropolymers [1, 2]. Keratins play important roles in mechanical integrity, cell signaling, and in protection against stress [3–6]. This is evidenced by keratin mutations that cause skin disease or predispose to liver disease in humans, and multiple similar disease associations have been reported by keratin mutations in mouse models [7, 8] (www.interfil.org). During various types of stress, resident keratins and *de novo* keratins are upregulated on both mRNA and protein level [5]. Upregulation of simple epithelial keratins has been shown, for example after griseofulvin-induced injury in mouse liver [9], caerulein- and choline/methionine-deficient diet injuries in pancreas [10] and high-fat lithogenic diet induced gallbladder injury [11]. Stress is also often accompanied with increased keratin phosphorylation and other post-translational modifications [3, 12, 13] and in patients with liver cirrhosis, increased keratin phosphorylation correlates to disease [14].

The role of keratins in the intestine is poorly understood although they are emerging as important players in colonic function [15]. However, K8 knockout (K8^**-/-**^) mice develop an early Th2-type colitis [16] including epithelial hyperproliferation [17] and decreased apoptosis [18]. This phenotype resembles the human inflammatory bowel disease (IBD) ulcerative colitis and is similarly amendable to antibiotic treatment indicating an involvement of luminal microbiota [18]. Though keratin mutations have also been reported in some IBD patients, keratin mutations have not been clearly confirmed to predispose humans to IBD [2, 8, 19]. On molecular level, the K8^**-/-**^ mouse distal colon has mistargeted and altered levels of membrane proteins and ion transporters [20, 21] which leads to a decreased short circuit current, increased net sodium absorption, decreased chloride secretion and diarrhea [21]. Genetic background effects are likely to be involved in some of the keratin murine phenotypes [22], since 94% of K8^-/-^ mice in the C57B1/6 genetic background strain die *in utero* while in the FVB/n background 50% of mice escape this embryo lethality and develop the IBD-phenotype [23].

The K8-null heterozygote (K8^+/-^) mouse colon appears histologically normal with no apparent inflammation, but the sodium and chloride transport is disturbed, albeit not to the same extent as in the K8^-/-^ [21]. This intermediate phenotype in the K8^+/-^ mice led us to hypothesize that the amount of keratins may play a role in protection against stress. In this study, we further characterize the K8^+/-^ colonic phenotype and report that K8^+/-^ express approximately 50% less keratins compared to K8^+/+^ mice leading to increased epithelial thickness and an increased susceptibility to experimental colitis.

## Materials and Methods

### Mice and ethics statement

K8^-/-^, K8^+/-^ and K8 wild type (K8^+/+^) mice in the FVB/n background were generated by breeding of K8^+/-^ mice and genotyped as previously described [17]. Animal studies were performed on sex- and age-matched littermates. All animal experiments were approved by the Animal Experimental Board in Finland (ESAVI_1197–04.10.07_2013) and conformed to the legal acts, regulations and requirements set by the European Union concerning protection of animals used for research.

### Antibodies

Primary mouse antibodies used for western blotting and immunostaining were rat anti-K8 and rat anti-K19 (Troma I respectively Troma III, Hybridoma bank, Iowa, USA), mouse anti-K7 (RCK 105, Abcam, Cambridge, UK), mouse anti-K20 (IT-Ks 20.10, Progen, Frankfurt, De), rat anti-K18 (Troma II, Hybridoma bank, Iowa, USA) [24], mouse anti-tubulin (Sigma, Munich, Germany), rabbit anti-caspase-7 and anti-cleaved caspase-7 (Cell Signaling, Danvers, MA, USA), rabbit anti-MPO (Thermo Scientific, Waltham, MA, USA) and rat anti-Hsc70 (Stressgen, Victoria, Canada). The secondary antibodies used for staining were Alexa 488 or Alexa 546 anti-mouse, Alexa 488 anti-rat and Alexa 488 anti-rabbit antibodies (Invitrogen, Carlsbad, CA, USA). The secondary antibodies used for western blotting were: anti-mouse HRP (GE Healthcare, Little Chalfont, UK), anti-rabbit HRP (Cell Signaling, Danvers, MA, USA) and anti-rat HRP (GE Healthcare, Little Chalfont, UK) antibodies. Nuclei were stained with Draq5 (Cell Signaling, Danvers, MA, USA) or Dapi (Invitrogen Carlsbad, CA, USA). Antibodies used for FACS analysis were anti-CD4-FITC and anti-CD49d-PE or anti-L-selectin-PE (Immunotools, Friesoythe, Germany).

### Sample collection, histology, crypt length measurement, immunofluorescence and microscopy

Mice were euthanized by CO_2_ inhalation and the colon was excised. The colon was divided into proximal (PC) and distal colon (DC), and further divided into 4 parts. For histology, samples were fixed in 4% paraformaldehyde (PFA), in phosphate buffer saline (PBS, pH 7.4), and subsequently stained with Hematoxylin and Eosin (H&E). Samples were fresh frozen and embedded in Optimal Cutting Temperature compound (OCT) (Sakura Finetek, Netherlands) for immunostaining, collected in RNA later (Qiagen, Limburg, Netherlands) for mRNA analysis, and collected in liquid nitrogen for protein analysis. The OCT samples were further cryosectioned (6 μm) and fixed with acetone at -20°C. Immunofluorescence staining on sections was performed using the antibodies listed above. Images were captured using a Zeiss LSM780 (Jena, Germany) and Leica TCS SP5 (Wezla, Germany) confocal microscopes. Images were compiled using Adobe Illustrator and Adobe Photoshop (San Jose, CA, USA) software. Crypt lengths in PC and DC from K8^+/+^, K8^+/-^ and K8^-/-^ mice were measured from digital photographs of H&E stained colon sections taken with a Zeiss Axiovert 200M (Jena, Germany) microscope and processed using ImageJ software (National Institutes of Health, USA). The crypt lengths are expressed as mean length ± SD, calculated based on 97–417 crypts from n = 3 animals / genotype.

### SDS-Page, Western blotting and quantification

Total lysates of intestinal tissues were prepared by homogenization in sample buffer containing 0.187M Tris-HCl pH 6.8, 3% sodium dodecyl sulphate (SDS) and 5mM ethylene diamine tetra acetic acid (EDTA). Protein concentration was measured using a Pierce BCA protein assay kit (Thermo Scientific, Waltham, MA, USA) and equal amount of protein was loaded and separated by SDS polyacrylamide gel electrophoresis, transferred to a polyvinylidene fluoride membrane and immunoblotted with indicated antibodies. Proteins were visualized using Western Lightning Chemiluminescence (Perkin-Elmer, Waltham, MA, USA). Protein signals were quantified from scanned films using ImageJ (National Institutes of Health, USA) by normalizing to Hsc70.

### High salt extraction and Coomassie staining

High salt extraction (HSE) was preformed according to a previously described protocol [24]. Briefly, tissue samples from PC, DC, liver and small intestine, were homogenized in Triton X100 buffer using a tissue homogenizer (TissueRuptor, Quiagen, Hilden, Germany). The samples were centrifuged and the pellet was collected. The pellets were manually homogenized in high salt buffer containing 10 mM Tris-HCl, 140 mM NaCl, 1.5 M KCl, 5 mM EDTA, 0.5% Triton X100, 1 mM phenylmethylsulfonyl fluoride (Sigma-Aldrich, St. Louis, MO, USA) and protease inhibitor mix (Complete, Roche, Mannheim, Germany), then mixed for 30 minutes at 4°C and re-pelleted. The pellets were washed in 5 mM EDTA/PBS buffer by manual homogenization, centrifuged to pellet, then re-suspended in Laemmli sample buffer. Samples were separated on a 10% acrylamide gel and stained using Coomassie Brilliant Blue.

### Reverse Transcriptase-PCR

Total colon RNA was isolated from colon epithelial scrapings obtained by scraping the mucosa with a microscope slide. RNA was isolated using an RNeasy mini kit (Qiagen, Netherlands). Contaminating DNA was removed with DNase I enzymes (Promega, WI, USA) and the RNA quality was analyzed in a 1% agarose gel. 1 μg of each RNA sample was synthesized by reverse transcription to cDNA using a transcription kit (Promega, WI, USA). Target genes were amplified using specific primers (S1 Table) and KAPA probe Fast ABI Prism qPCR mix (Kapa Biosystems, MA, USA). qPCR was performed with Step One Plus Real-Time PCR system (Applied Biosystems, CA, USA). Gene expression levels were normalized to the housekeeping gene β-actin. Each cDNA was tested in triplicate and the amplification was analyzed using a 1% agarose gel.

### BrdU labeling and quantification

Mice were injected by intraperitoneal (i.p.) route with 200 μl 5 mg/ml 5-bromo-2'-deoxyuridine (BrdU), BD Pharmingen, San Diego, CA, USA) in PBS, and sacrificed 4 hours after injection. Proliferative cells were stained with a BrdU detection kit (BD Pharmingen, San Diego, CA, USA) according to the manufacturer’s protocol with the modification that sections were incubated in 0.5% Borax solution (Sigma Aldrich, St. Louis, MO, USA) and 0.3% H_2_O_2_ (Sigma Aldrich, St. Louis, MO, USA) before the antigen retrieval and primary antibody incubation. The fraction of proliferative cells was quantified by counting the number of proliferative cells in relation to the total number of cells in the colonic crypts. Two mice from each genotype were quantified and 15–20 crypts / mouse were analyzed.

### *Ex vivo* colon culture

Apoptosis of colonic tissue was measured according to the method described by [18]. Briefly, 5x5 mm colon pieces (DC and PC combined) were frozen immediately after collection (control) or incubated in cell medium (RPMI-1640 with 10% fetal calf serum (FCS), and penicillin/streptomycin) for 1 hour at 37°C to induce anoikis/apoptosis. The samples were washed with PBS immediately and stored in liquid nitrogen. The samples were homogenized and the protein concentrations were determined and further analyzed by western blotting as described above.

### *In vivo* imaging of RONS

Reactive oxygen and nitrogen species (RONS) were detected by *in vivo* imaging using the luminescent probe L-012 (Wako Chemical, Neuss, Germany) as recently described [25]. Briefly, L-012 was administered at 20 mg/kg by i.p. injection to isoflurane (1.5–2.5%) anesthetized mice. The L-012 solution was prepared fresh prior to each experiment by dissolving 2 mg L-012/ml in sterile PBS protected from light. Mice were directly moved into the imaging chamber (IVIS Lumina II imaging system, Xenogen/Caliper Life Sciences, USA) and the number of photons produced by L-012 was recorded in the anesthetized mice for 30 minutes with 1 minute exposure time. The Living Image (Waltham, MA, USA) software automatically co-registers and overlays the bioluminescence images with a photographic image of the mouse.

### Lamina propria cells isolation and flow cytometry

Colon lamina propria cells were isolated from K8^+/+^ and K8^+/-^ mice according to a method previously described by Alam et al. [26]. In brief, colons were excised, washed in Hank’s buffered saline solution (HBSS) (Sigma-Aldrich, St. Louis, MO, USA) supplemented with 10 mM Hepes (Sigma-Aldrich, St. Louis, MO, USA), cut into 5 mm^2^ pieces and incubated in HBSS/Hepes buffer containing 2% FCS and 2 mM EDTA pH 8, 3 time for 15 minutes at 37°C. The colon pieces were then digested with collagenase VIII (Sigma-Aldrich, St. Louis, MO, USA), 100 U/ml in RPMI-1640 supplemented with 10 mM Hepes and 10% FCS for 1 hour, under constant stirring, at 37°C. Digested colon pieces were mixed into HBSS/Hepes buffer containing 40% Ficoll PM 400 solution (Amersham Biosciences, Little Chalfont, UK), overlaid with a 70% Ficoll gradient and centrifuged at 1950 rpm, for 20 minutes at 4°C, after which the isolated lamina propria cells were collected from the 40% / 70% Ficoll interphase and washed twice in RPMI-1640 before use. The isolated lamina propria cells were stained with indicated antibodies diluted in PBS supplemented with 2% FCS, for 30 minutes at 4°C. The cells were washed in PBS, fixed with 1% PFA and analysed using fluorescence-activated cell sorting (FACS) FACS Calibur flow cytometer (BD Biosciences, Franklin Lakes, NJ, USA) and Flowing Software 2.5 (Cell Imaging Core, Turku Centre for Biotechnology, Turku, Finland).

### Dextran sulphate sodium colitis

Experimental colitis was induced in K8^+/+^ and K8^+/-^ mice by giving 2% or 5% dextran sulphate sodium (DSS; TdB consultancy, Uppsala Sweden; 40 000 Da) in autoclaved drinking water for 7 days, followed by autoclaved water for 4 or 2 additional days for the 2% and 5% DSS experiment, respectively. In a different experiment, 3% DSS was administered in the drinking water for 5 days, followed by autoclaved drinking water for 7 days. The 5% DSS treatment lead to the most severe disease and in some mice lethality. In all DSS experiments, to ensure humane treatment, mice were observed 1–3 times per day (during days 1–7) and for the 5% DSS experiment 3 times per day, then with 6–8 hour intervals during days 8 to 10 to monitor mouse weight and overall health. Mice were sacrificed by CO_2_ inhalation if their body weight was reduced below 20% of the body weight at the start of the experiment according to the approved protocol. Mice that died suddenly between monitoring hours (but had lost less than 20% body weight) and mice that were moribund and sacrificed due to 20% loss of body weight were considered dead in the survival analysis. Mouse appearance such as fur condition and mobility in the cage were also monitored, and it was ensured that mice had access to water and food. No analgesics were used in the study. For the chronic DSS experiment, mice were administered 2.5% DSS for 7 days, followed by autoclaved water for 14 days, then a second round of 2.5% DSS for 7 days, followed by normal water until mice were sacrificed 7 or 17 days after the second DSS administration. Control mice were given normal water throughout the experiment. Day 1 denotes the baseline situation and when DSS-treatment was started. Drinking bottles were replaced with fresh DSS solution on day 3 and 5. Mice were weighed, observed for blood and loose stools daily or on indicated time points during the experiment [27]. The colitis score from H&E-stained samples was determined by two scientists in a blinded fashion according to the method reported [28]. The severity of the diarrhea was assessed by scoring stool consistency using the following key: solid = 1, soft = 2, slightly watery = 3, and watery = 4. Rectal bleeding was scored using the following key: none = 0, traces of blood = 1, blood mixed-stool = 2, congealed blood around rectal areas = 3, fresh bleeding at rectal areas = 4. Disease activity index (DAI) is a score where stool consistency, bleeding score and body weight loss is combined into one value determining how severely the mice are affected by colitis. The DAI was calculated as: 1 point for every 5% of body weight loss; stool consistency score is scored as: 0) for solid pellets, 1) soft pellets, 2) slightly loose and 3) liquid and the bleeding score defines the presence of blood: 1) blood found in stool pellets and 2) blood found at anus either clotted or fresh.

### Statistical analysis

Statistical analysis was performed by using student t-test (% body weight change, stool hydration, colon length, occult blood score, crypt length), Mann-Whitney U test (*in vivo* imaging), One way Anova and Bonferroni post hoc test (mRNA, BrdU and protein levels), and Kaplan-Meier test (survival) using GraphPad Prism 5 software (La Jolla, CA, USA).

## Results

### Heterozygous deletion of K8 results in 50% less keratin protein and K8 mRNA in the colonic epithelium

**Fig 1:**
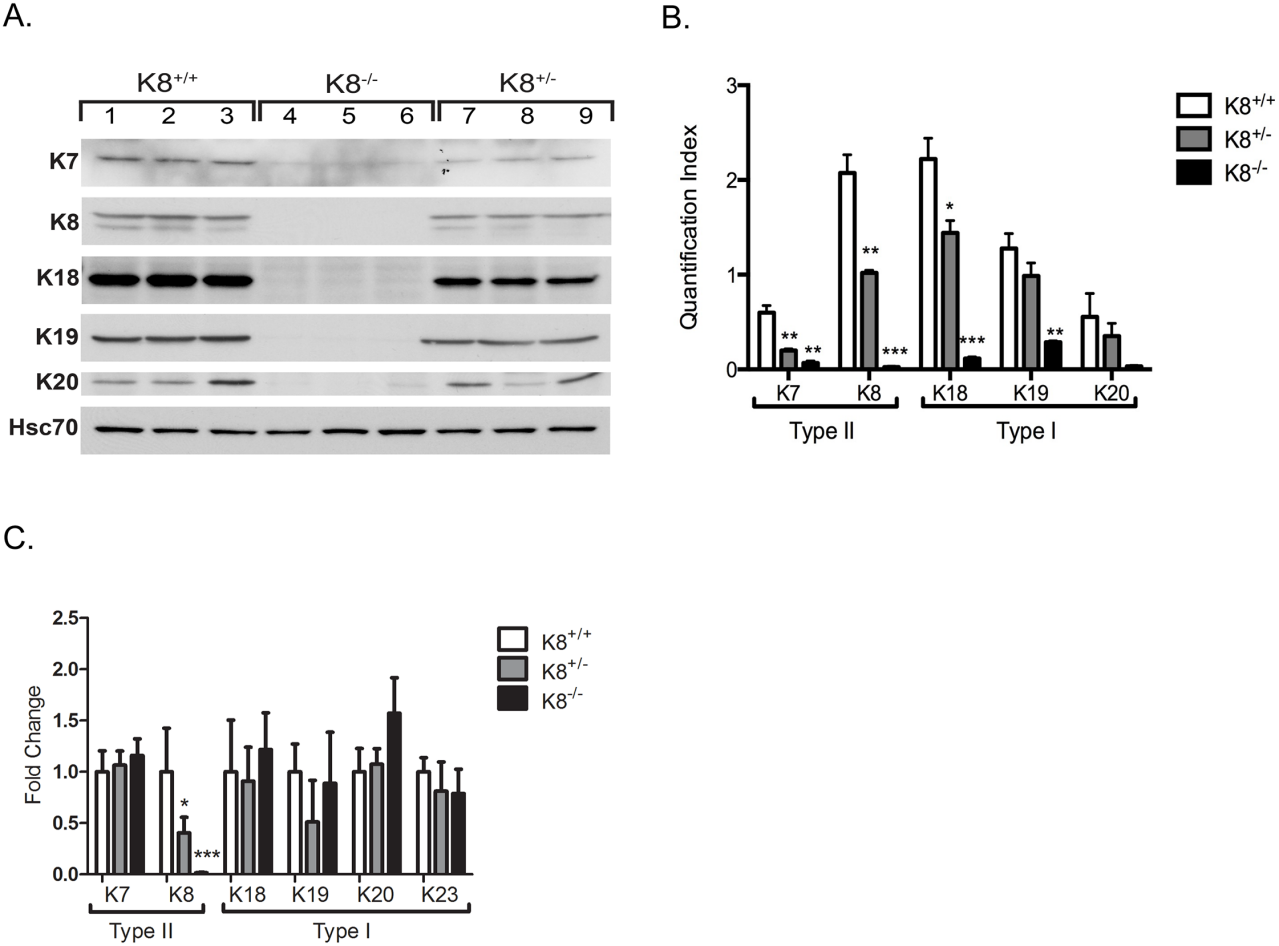
K8^+/-^ mice have decreased levels of colonic keratins and K8 mRNA. A. Total lysates of colon from K8^**+/+**^, K8^**+/-**^ and K8^**-/-**^ mice (n = 3) were analyzed for the amount of K8, K18, K19 and K20 using SDS-PAGE and western blotting. Equal amount of protein was loaded to each well and Hsc70 was used as loading control. B. Keratin protein expression was quantified using ImageJ software. C. RT-PCR was performed to measure keratin mRNA levels in total RNA isolated from colon scrapings. Target genes were amplified using specific primers and KAPA probe Fast ABI Prism qPCR mix. Gene expression levels were normalized to the housekeeping gene β-actin. * = P < 0.05. Results are given as means ± SEM.

To assess whether K8^+/-^ mice, which have only one K8 allele, express decreased levels of keratins in the colon, western blots and RT-PCR for keratins K7, K8, K18, K19 and K20 were performed and keratin levels in K8^+/-^ mice were compared to sex- and age-matched littermate K8^+/+^ and K8^-/-^ mice (Fig 1). At the protein level, the K8^+/-^ colon has 50% less K8 compared to the K8^+/+^ colon (Fig 1A and 1B). The second type II keratin, K7 and type I K18 were also significantly decreased in K8^+/-^ colon, and to a lesser extent also K19 and K20 compared to K8^+/+^. RT-PCR analysis showed a similar ~50% decrease of K8 mRNA in the K8^+/-^ colon and a complete loss in K8^-/-^, compared to K8^+/+^, while mRNA for the other keratins were unaltered (Fig 1C). High salt extraction of colonic keratins showed that K8 and K19 are the main type II and type I keratins in colon, respectively, and that no compensatory keratin protein or other major protein precipitated with high salt is increased in K8^+/-^ and K8^-/-^ mice (S1 Fig).

### The distribution of keratins in K8^+/-^ colon is unaltered except for K7, which is expressed higher up in the K8^+/-^ crypts

**Fig 2:**
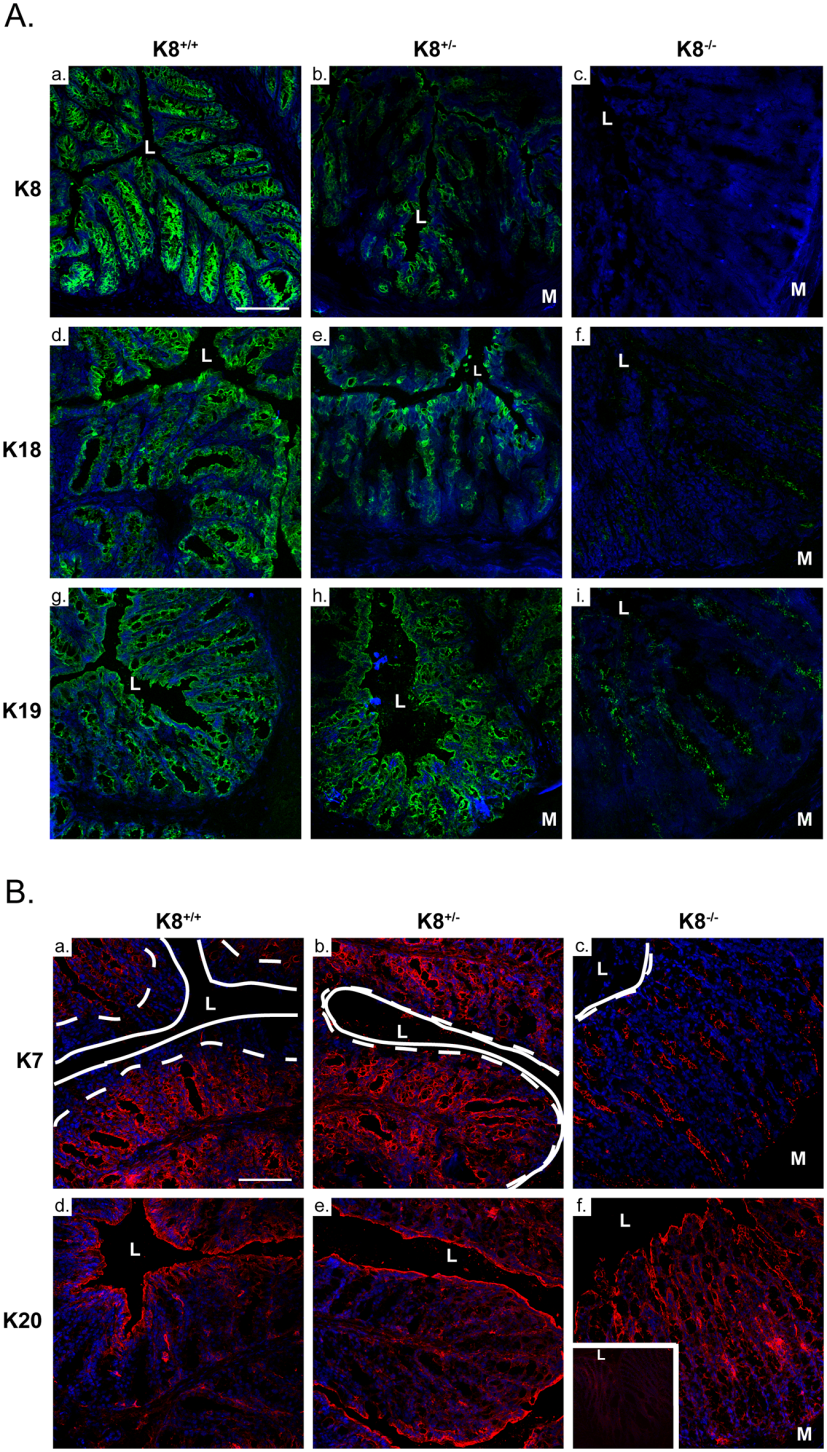
K8 deletion results in increased expression of K7 in the top of colonic crypts. Fresh frozen sections from K8^**+/+**^, K8^**+/-**^ and K8^**-/-**^ mice (n = 3) were fixed with acetone and stained for A. K8, K18, K19, and B. K7, K20. The distribution of K7 in K8^**+/-**^ and K8^**-/-**^ crypts is extended to the top of the crypts (B: b, c) compared to K8^**+/+**^ where K8 is the only type II keratin in the top epithelial cells (area between dotted and solid line, B: a and A: a, b). Scale bar = 100 μm. L = lumen; M = muscle. Nuclei = blue. Insert in Bf. shows signal from secondary antibody alone.

The distribution of colonic keratins was analyzed by immunofluorescence staining (Fig 2A and 2B) using specific primary antibodies for each keratin. The distribution of K8, K18, K19 and K20 were unaltered in the K8^+/-^ colon (Fig 2A and 2B), although the decreased protein levels quantified in Fig 1 were clearly distinguishable under these carefully comparable confocal scanning settings (Fig 2A and 2B). K7 which normally is expressed mainly in the bottom part of the crypts, was however, found to be expressed in all cells throughout the crypt in the K8^+/-^ colon (Fig 2Ba–2Bc) suggesting that K7 compensates in the absence of K8 in the cells close to the luminal region. The keratin distribution in K8^+/+^, K8^+/-^ and K8^-/-^ colon is schematically summarized in S2 Fig.

### K8^+/-^ mice show a moderate colonic hyperproliferation phenotype but no spontaneous intestinal inflammation

**Fig 3:**
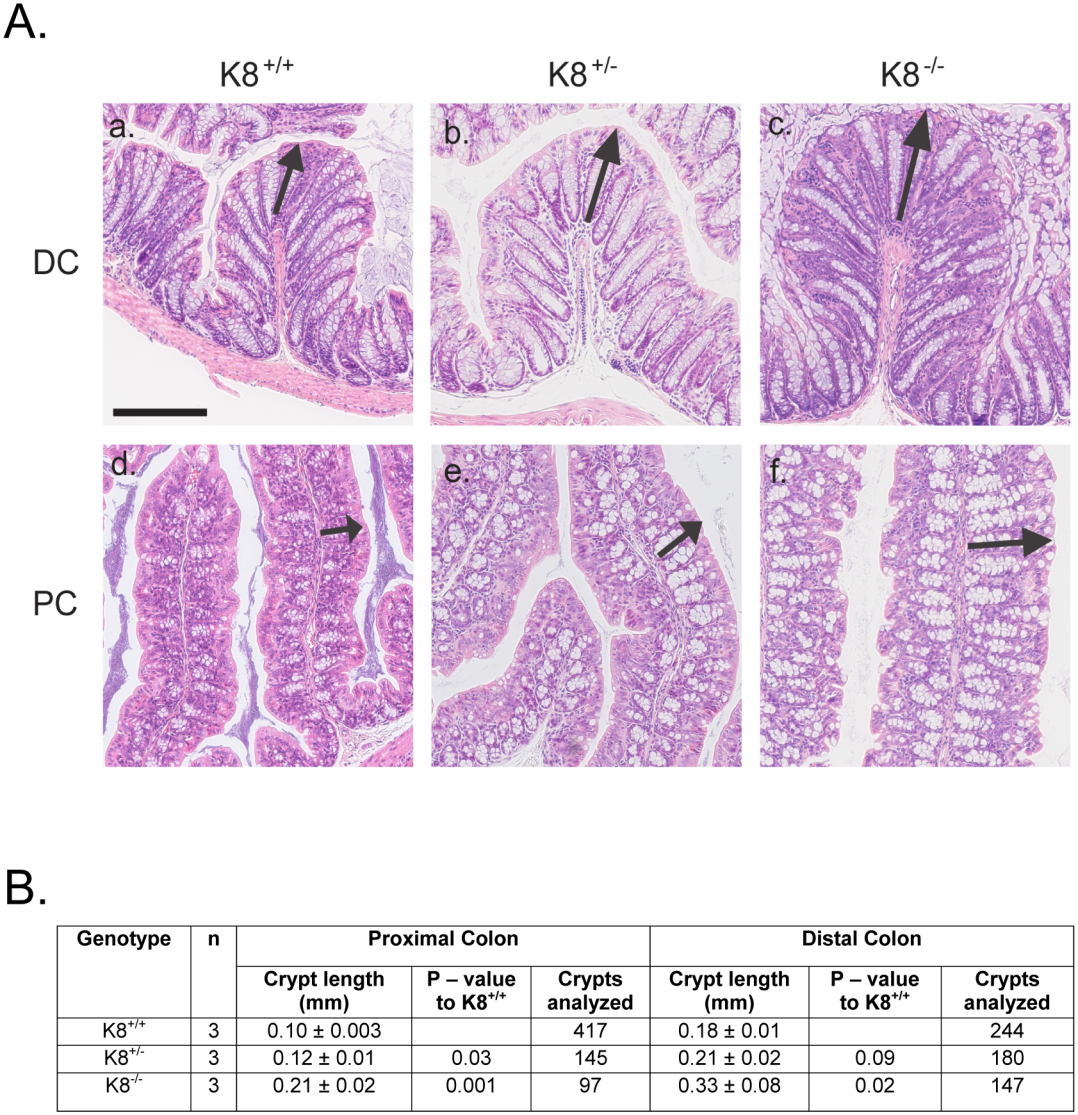
K8^+/-^ mice have increased crypt length. A. The K8^**+/-**^ baseline morphology was studied by H&E staining of the distal colon (DC) and proximal colon (PC) and the crypt lengths were measured (black arrows). B. Crypt lengths in PC and DC were measured from digital photographs of H&E stained colon sections (n = 3) taken with Zeiss Axiovert 200M microscope and processed using ImageJ software. The crypt lengths are expressed as mean length ± SD, calculated based on crypts from 3 animals / genotype.

**Fig 4:**
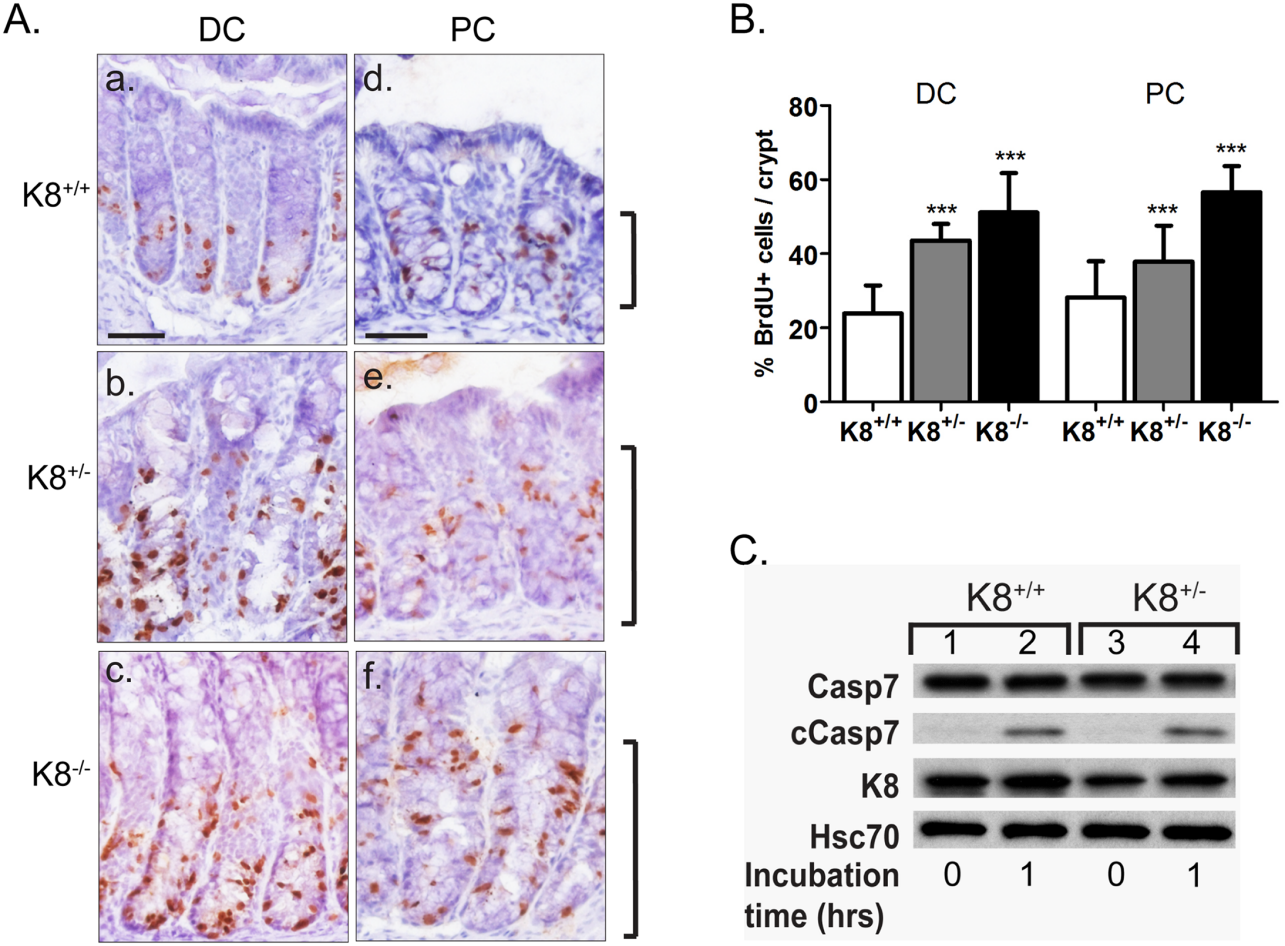
Increased number of proliferating cells in K8^+/-^ but unaltered anoikis of colonic crypts. A. and B. Mice were injected with BrdU (Bromodeoxyuridine, 5-bromo-2-deoxyuridine) i.p. 4 hours before sacrifice and proliferative cells were stained with anti-BrdU and quantified. Proliferating cells were significantly increased in both K8^**+/-**^ and K8^**-/-**^ mice DC (distal colon; A. b, c) and PC (proximal colon; A. e, f) compared to K8^**+/+**^ mice (A. a, d). Scale (a, d) = 50 μm. Brackets indicate the proliferative cell zone at the bottom of the crypt. B. The fraction of proliferative cells was quantified by counting the number of proliferative cells in relation to the total number of cells in the colonic crypts. Increased number of BrdU positive cells directly indicates the hyperproliferation in K8^**+/-**^ and K8^**-/-**^ colon. *** p<0.001. C. The level of apoptosis was assessed from K8^**+/-**^ and K8^**-/-**^ colon lysates by immunoblotting for cleaved caspase 7 (cCasp7) directly after excising from the mouse or after 1 hour of incubation at 37°C in cell culture medium. Hsc70 and caspase 7 (Casp7) were used as loading controls.

We next examined the K8^+/-^ colon histology to assess whether the decreased K8 expression causes histopathological changes. H&E staining showed that the crypts in K8^+/-^ DC and PC were significantly longer (Fig 3A) compared to K8^+/+^, but shorter than the previously described K8^-/-^ hyperplastic crypts (Fig 3A) [17, 21]. The average crypt length in K8^+/+^ and K8^+/-^ proximal colon were 0.10 ± 0.003 mm and 0.12 ± 0.01 mm, respectively, and in K8^+/+^ and K8^+/-^ distal colon 0.18 ± 0.01 mm and 0.21 ± 0.02 mm, respectively. The crypt lengths in K8^-/-^ mice were 0.21± 0.002 mm and 0.33 ± 0.08 mm (for PC and DC, respectively; Fig 3B). To test whether the longer crypts were related to increased proliferation, mice were injected with the proliferation marker, BrdU, which marks dividing cells. The BrdU staining indicated a wider zone of proliferative cells in the K8^+/-^ proximal and distal colon (Fig 4A) compared to K8^+/+^. Quantification revealed that K8^+/-^ colon had a significantly higher percent of BrdU-positive cells/crypt, compared to K8^+/+^, but fewer compared to the K8^-/-^ colon (Fig 4B). In contrast to the resistance to anoikis (apoptosis) we have described in K8^-/-^ [18], no difference in anoikis was noticed in the K8^+/-^ colon when assayed for cleaved caspase 7 one hour after ex vivo culture (Fig 4C).

**Fig 5:**
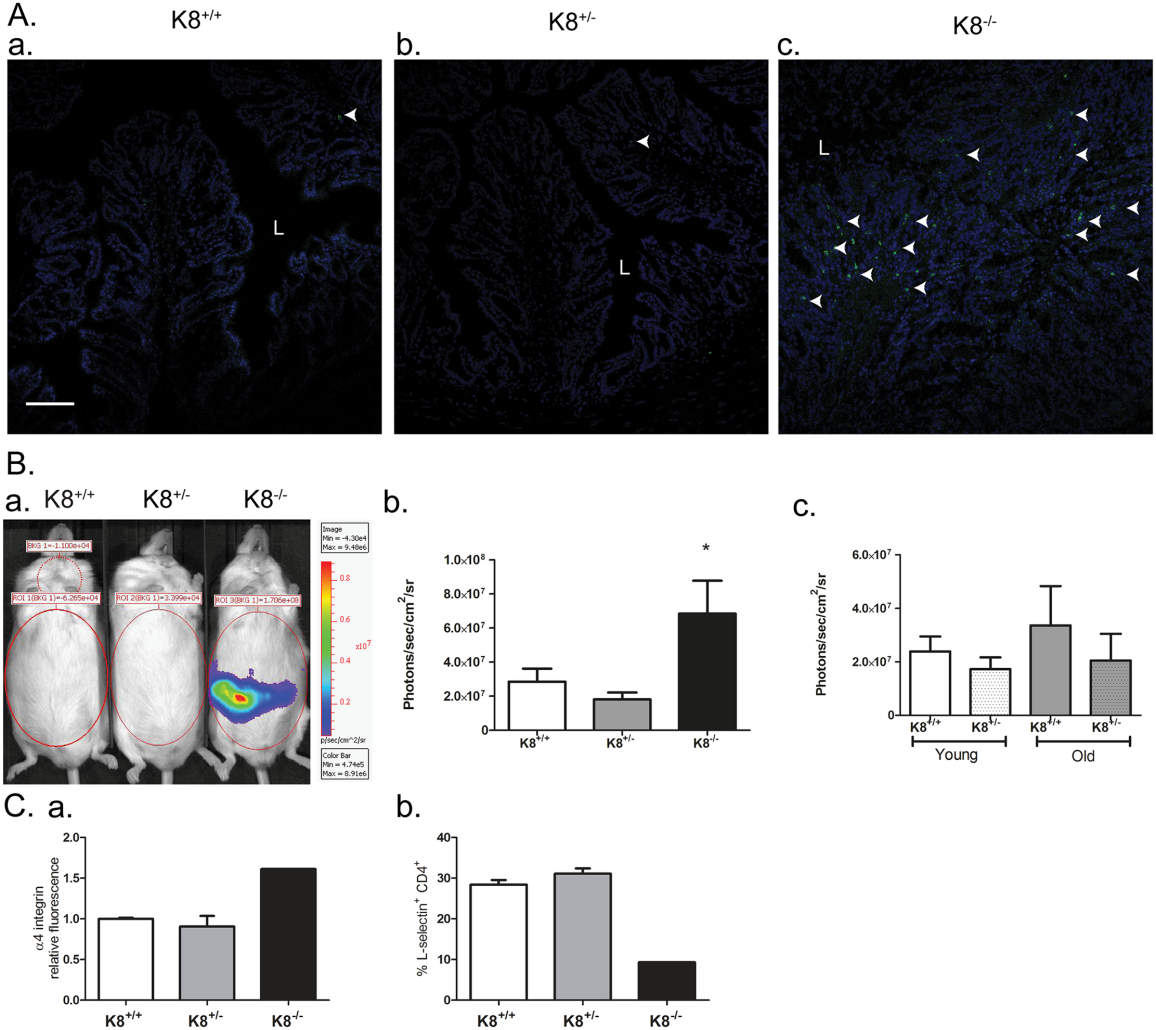
K8^+/-^ mice have no baseline inflammation in contrast to K8^-/-^. A. Cryosections from K8^**+/+**^, K8^**+/-**^ and K8^**-/-**^ mice (n = 3) were stained for myeloperoxidase (MPO). White arrows indicate MPO positive cells. Scale bar: 100 μm; L = lumen, M = muscle. B. *In vivo* imaging for RONS. B.a. For *in vivo* imaging of inflammation, mice were injected i.p. with L-012 and luminescent signals obtained by an IVIS camera (Ba). The abdominal chemiluminescence signals were quantified and shown as average ± SD in adult mice (Bb), and young versus old mice (Bc). C. The colonic lamina propria (LP) cells were isolated from K8^**+/+**^ and K8^**-/-**^ mice, stained with anti CD4-FITC and anti-CD49d-PE or anti-L-selectin-PE and further analysed using a flow cytometer.

To assess whether K8^+/-^ mice may be prone to spontaneous colitis similar to K8^-/-^ [16, 18], the K8^+/-^ colon was analyzed for signs of inflammation. Myeloperoxidase (MPO) is an enzyme secreted by neutrophils at the site of inflammation [29] and is widely used as a marker of murine colitis. MPO was clearly increased in the K8^-/-^ colon (Fig 5Ac) while K8^+/-^ MPO levels were comparable to those in K8^+/+^ (Fig 5Aa and 5Ab) indicating no major inflammation. To monitor oxidative stress in K8^+/-^ mice, we used a recently established *in vivo* imaging method that detects RONS using the chemiluminescent probe L-012 [25, 30]. K8^+/-^ mice showed abdominal RONS signals similar to K8^+/+^ mice, whereas K8^-/-^ had high signals (Fig 5Ba and 5Bb). The RONS signals did not increase in aging K8^+/-^ mice (Fig 4Bc). In contrast to the clear inflammatory profile of the K8^-/-^ colon lamina propria T-cells, the K8^+/-^ colon T-cells were very similar to those in K8^+/+^ colon in terms of number of T-cells (not shown), percentage of naïve (L-selectin positive) to effector T-cells (L-selectin negative) and integrin α4 expression (Fig 5Ca and 5Cb). Furthermore the colon length is similar in K8^+/+^ and K8^+/-^ mice, in contrast to K8^-/-^ mice, which have a shorter colon (S2 Table). Taken together, the decreased levels of K8 in the K8^+/-^ mouse colon leads to an intermediate phenotype with longer crypts, but without inflammation.

### K8^+/-^ mice are more sensitive to DSS-induced experimental colitis

**Fig 6:**
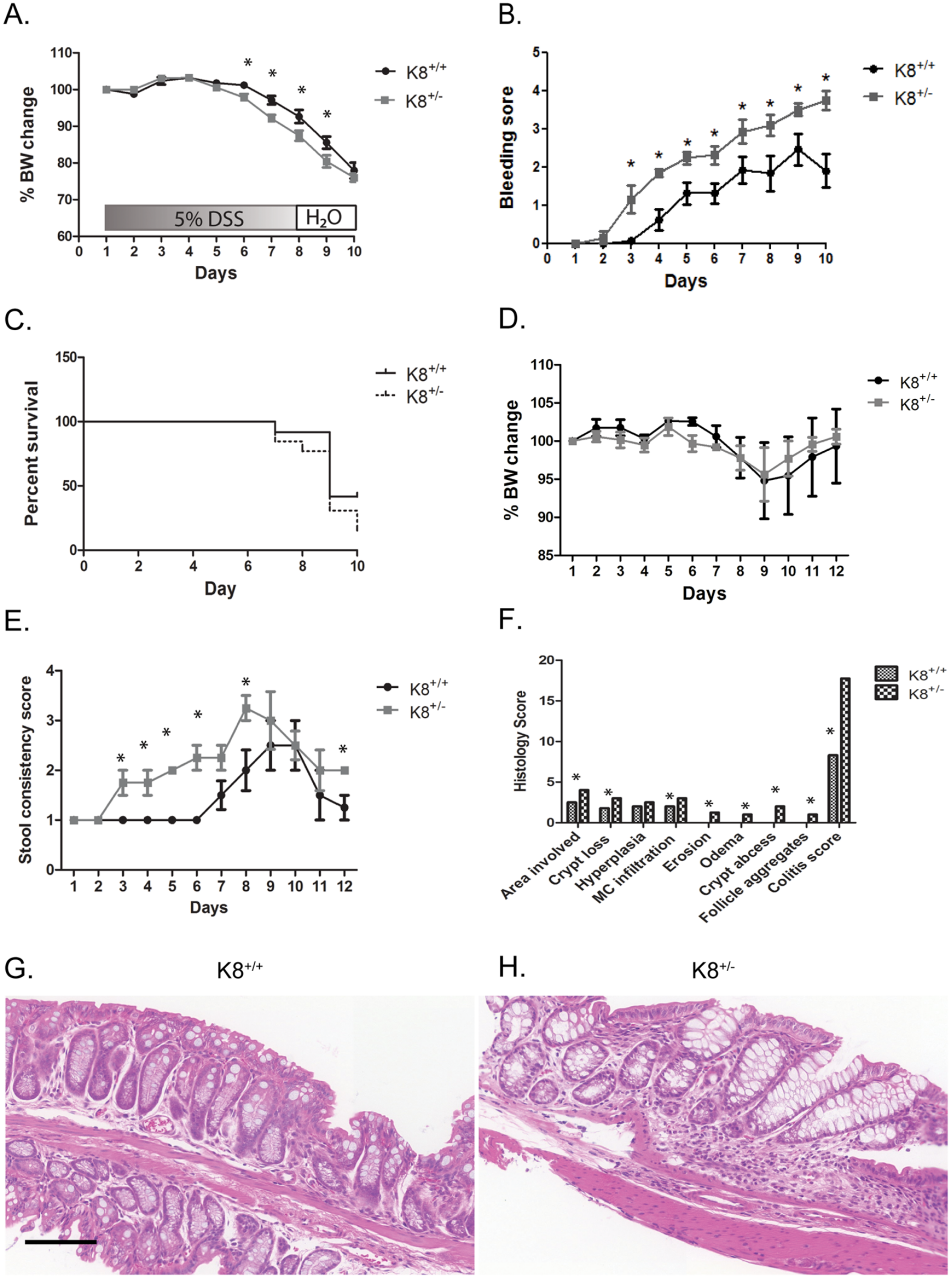
Increased susceptibility of K8^+/-^ mice to experimental colitis. Mice were given 5% DSS in drinking water for 7 days followed by regular water for 2 days and assessed for body weight change (A), bleeding score (B) and survival (C). (n = 12–13, p = < 0.05 on day 10). D. 3% DSS in autoclaved drinking water mice were administered up to day 5 followed by drinking water up to day 12 (D-H). *p < 0.05. F. Longitudinal sections from colon were stained with H&E staining and (G and F), and a histological score was determined. Scale bar: 100 μm.

We hypothesized that the decreased levels of keratins may render K8^+/-^ mice more susceptible to colonic stress. To test this, mice were treated with different regimens of DSS in the drinking water followed by different recovery periods to induce acute or chronic disease [27]. An acute regimen of 5% DSS for 7 days plus 2 days with DSS-free drinking water, resulted in body weight loss, moderate to severe diarrhea and rectal bleeding in both K8^+/-^ and K8^+/+^ mice (Fig 6A–6C). The body weight loss was slightly but significantly greater in K8^+/-^ mice compared to K8^+/+^ mice on days 6–9 (Fig 6A) and K8^+/-^ mice had significantly more severe rectal bleeding from day 3 onwards (Fig 6B). The high dose of 5% DSS resulted in lethality after day 7 which was higher in K8^+/-^ compared to K8^+/+^ mice: on day 10, 8% of K8^+/-^ survived compared to 42% of K8^+/+^ mice (p < 0.05 on day 10; Fig 6C). In both K8^+/+^ and K8^+/-^ mice, 5% DSS induced a significant shortening of the colon compared with healthy K8^+/+^ and K8^+/-^ mice, respectively, while no difference in colon length was noted between treated K8^+/+^ and K8^+/-^ mice (S2 Table).

To induce acute but less severe colitis, mice were administered 3% DSS in autoclaved drinking water for 5 days followed by autoclaved water up to day 12 (Fig 6D–6H). As expected, the 3% DSS regimen compared to 5% DSS resulted in a less dramatic body weight change which was only significantly different between K8^+/-^ and K8^+/+^ on day 7 (Fig 6D). However, K8^+/-^ mice had significantly higher stool consistency score compared to K8^+/+^ mice (Fig 6E; on days 3–6, 8, 12) and the DAI was significantly higher during days 3–6 between the genotypes (not shown). Histological scoring showed a higher colitis score for K8^+/-^ than K8^+/+^ (Fig 6F) at the end of the experiment on day 12. In a small-scale chronic DSS experiment (two rounds of 7 days with 2.5% DSS + with a 2 week brake with autoclaved water), K8^+/-^ mice also were more sensitive and displayed slightly worse histology, higher average DAI, more MPO-positive cells, slightly more apoptosis (S3A–S3C Fig) and shorter colon length (S2 Table) compared than K8^+/+^. The DSS data also suggest that K8^+/-^ mice respond faster to DSS (Fig 6B and 6E), and recover more slowly from the DSS (Fig 6B and 6E, S3A Fig) compared to K8^+/+^. Taken together, K8^+/-^ mice are more susceptible to DSS-colitis than littermate K8^+/+^.

## Discussion

We have here characterized the pathological, immunological and stress-response phenotype of the K8^+/-^ mouse colon where one of the two K8 alleles have been inactivated (Table 1). The hitherto reported colonic phenotype of this mouse is normal-appearing histology, but a sodium and chloride transport-defect intermediate between control K8^+/+^ and the nearly keratin-free K8^-/-^ colon [17, 21]. We here show that heterozygous deletion of K8 results in a 50% decrease in K8 mRNA and K8 protein in the colonic epithelium. The remaining keratins are spatially normally distributed in the colonic crypt, except for K7 which is expressed higher up in the crypts compared to K8^+/+^, but not increased on overall protein level. The K8^+/-^ mice have an intermediate colonic hyperproliferation phenotype, but in contrast to the K8^-/-^ colon tissue [16], they lack any signs of spontaneous intestinal inflammation. K8^+/-^ mice have, however, increased sensitivity and delayed recovery from DSS-induced experimental colitis suggesting that reduced keratin levels significantly compromise stress protection and the regenerative capacity of colonic epithelial cells.

**Table 1:** Summary of the K8^+/-^ colonic phenotype.

Phenotype	K8^+/+^	K8^+/-^	K8^-/-^	Reference
Keratin levels	++++	++	+	This study
Crypt length	+	++	++++	This study
Colon length	Normal	Normal	Shorter	This study
Abnormal Na/Cl transport	-	++	++++	[21]
Basal apoptosis/anoikis	Normal	Normal	Decreased	[18], this study
Spontaneous colitis/inflammation	-	-	++++	[16, 17], this study
Experimental colitis	+	++	ND	This study

ND, not determined.

Apart from the 50% decreased levels of K8, K8^+/-^ colonic crypt epithelial cells contain all colonic keratins including K7, K18, K19 and K20 at normal mRNA levels, but have decreased average protein levels of all keratins and significantly decreased levels of K7 and K18. Together this data suggest that keratin mRNA are transcribed independent of K8 mRNA, while sufficient K8 mRNA and the presence of K8 as a type I keratin partner is needed for protein and filament stabilization. No other type II keratin mRNA was increased in a microarray comparison of K8^+/+^ and K8^+/-^ colonic crypt scrapings (data not shown). The decreased levels of K8^+/-^ keratins were seen both by western blotting and immunostaining, and the staining results further indicate that keratin levels are decreased evenly throughout the crypt. Interestingly, K7, which is normally expressed only at the base and middle regions of the colonic crypts, was expressed *de novo* higher up in the in K8^+/-^ crypts indicating that K7 is compensating for K8 in this specific tissue compartment where K8 is normally the only type II keratin. A similar *de novo* K7 expression was also seen in the K8^-/-^ colonocyte apical compartment which lacks cytoplasmic filaments altogether. Detecting K7 in this K8^+/-^ and K8^-/-^ cellular compartment is anticipated since type I keratins (K18, K19, K20) are present there by staining, requiring a type II keratin for protein and filament stabilization. The apical compartment of keratins is likely important for the integrity of this single layered epithelium as seen in the small intestine [20]. In the recently generated K7^-/-^ mouse, a similar 50% decrease in K7 mRNA and protein was noted in the K7^+/-^ bladder epithelium while the other keratin mRNAs were unaltered [31]. Interestingly, a small increase in K20 mRNA was described in the K7^-/-^ bladder and a similar slight, but not significant, elevation is noted in the K8^-/-^ colon. Our finding in the colon suggest that K8 levels are essential in regulating the protein levels of the other intestinal keratins, and that the clear gene dose-dependent difference in keratin protein levels, i.e. K8^-/-^ < K8^+/-^ < K8^+/+^, could be used as a controlled *in vivo* model to further study the effect of keratin levels in intestinal health.

Based on the above findings on decreased keratin levels in the K8^+/-^ colon and the emerging function of IFs and keratins as important players in cytoprotection from stress [5, 6, 32, 33], we analyzed for a possible tissue proliferation, apoptosis and inflammation phenotype in these mice. Careful histological analysis revealed that the K8^+/-^ colonic epithelium is significantly thicker than the K8^+/+^ mouse colonic epithelium due to increased epithelial cell proliferation, however, no decreased anoikis could be detected as in contrast to the more dramatically thickened K8^-/-^ epithelium [18]. Such increased proliferation has not to our knowledge previously been reported for the loss of one keratin allele and e.g. the K7^+/-^ bladder had no reported phenotype although the full knockdown of K7 bladder, similar to the K8 colon epithelia, is hyperproliferative [31]. Keratin function has been linked to proliferation/cell cycle as reviewed by Pan et al. [6] and e.g. knockdown of keratins is associated with increased proliferation in breast cell culture systems, and keratin overexpression correlates with decreased proliferation [34]. The signaling mechanisms behind how low keratin levels leads to increased proliferation needs further investigation but may involve keratin interaction with 14-3-3 or Akt signaling [6]. In contrast to the K8^-/-^ colitis phenotype [16] no signs of inflammation were detected in the K8^+/-^ using several different techniques. Together with the previously reported significant K8-dose dependent ion transport defect-phenotype [21], these data suggest that normal K8 levels are required to maintain intestinal homeostasis on proliferation and ion transport, while 50% of normal keratin levels are sufficient to protect from developing spontaneous colitis. Keratins can be considered stress proteins with many functions in common with the *bona fide* heat shock stress proteins, such as significant up regulation in response to various stresses, protein abundance, cytoprotection, and the linkage IF protein or heat shock mutations to several human diseases [5]. Our studies further support a function in stress-protection in the colon, since K8^+/-^ mice are more sensitive when stressed with the experimental colitis model DSS. K8^+/-^ mice also showed slower recovery from DSS suggesting an importance of keratins in both stress protection and recovery from stress. Whether the increased K8^+/-^ proliferation is linked to colitis susceptibility is unclear and has not been comprehensively studied in other models. Interestingly, deletion of ion transporters has been linked to increased colonic epithelial proliferation [35] and this keratin-ion transport link warrants further investigation. However, knockdown of the Wnt agonist Dkk, which leads to a similar basal increased proliferation, protects mice from DSS-induced colitis and recovery [36], indicating that these pathways are complex.

While a few IBD patients with keratin mutations have been described, it is currently unclear if keratin mutations subject humans to IBD [2]. Recently, however, it has been shown that K8 and K18 mutations disrupt the barrier function in colonic cell cultures [37]. Patients with keratin mutations that predisposed to liver diseases typically develop liver disease if subjected to a second stress that specifically affects the liver, such as drugs or alcohol [2]. In this sense, the K8^+/-^ colon with a rather mild baseline phenotype but increased susceptibility to the second stress by DSS, should serve as a good model to study keratins in the intestine without the effect of baseline colitis or overexpressing a keratin mutation, where the increased keratin levels may be protective.

## Supporting Information

S1 Fig K8 deletion does not lead to an increase of compensating proteins in the high salt cytoskeleton fraction of the colon. (DOCX)

S2 Fig Schematic overview of the keratin crypt distribution as a function of full or partial K8 deletion. (DOCX)

S3 Fig K8^+/-^ mice are more sensitive to chronic 2.5% DSS treatment. (DOCX)

S1 Table Forward and reverse primers used for RT-PCR. (DOCX)

S2 Table Colon length measurements in untreated and DSS-treated K8^+/+^ and K8^+/-^ mice. (DOCX)
