# Supplementary material for: The Amount of Keratins Matters for Stress Protection of the Colonic Epithelium
Source: PLoS One. 2015 May 22;10(5):e0127436. doi: 10.1371/journal.pone.0127436 (PMC4441500; doi:10.1371/journal.pone.0127436)
Supplement: S1 Fig — (DOCX) [file pone.0127436.s001.docx]

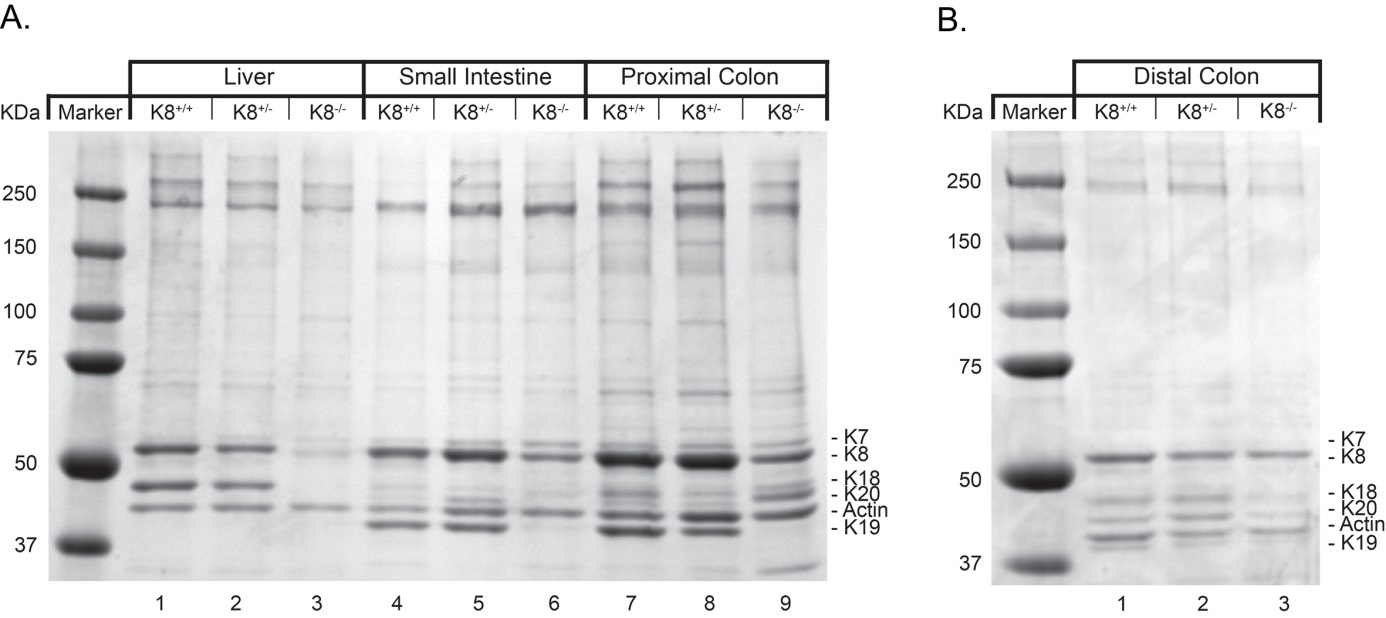


**Figure S1. K8 deletion does not lead to an increase of compensating proteins in the high salt cytoskeleton fraction of the colon.** Keratins from K8^+/+^, K8^+/−^ and K8^−/−^ (A) liver, small intestine, proximal and (B) distal colon were extracted by a high salt solution and separated by SDS-PAGE. The proteins in the gels were then stained by Coomassie brilliant blue. The specific keratin bands were verified by western blotting (not shown) and size (kDa). The liver samples serve as positive control for K8 and K18.
