# Supplementary material for: The Amount of Keratins Matters for Stress Protection of the Colonic Epithelium
Source: PLoS One. 2015 May 22;10(5):e0127436. doi: 10.1371/journal.pone.0127436 (PMC4441500; doi:10.1371/journal.pone.0127436)
Supplement: S2 Fig — (DOCX) [file pone.0127436.s002.docx]

**
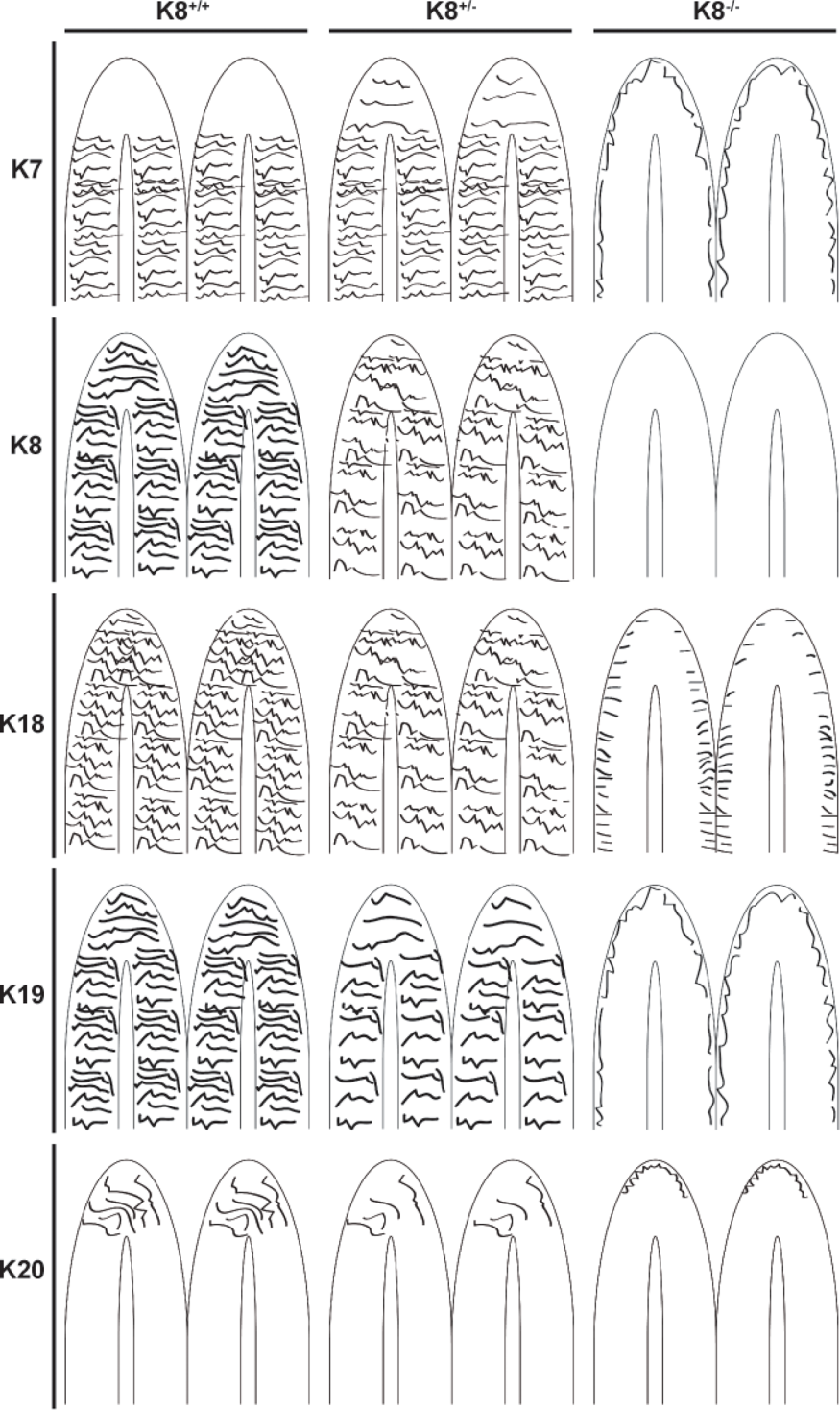
**

**Figure S2. Schematic overview of the keratin crypt distribution as a function of full or partial K8 deletion.** Each of the small individual images represents two crypts, where lumen is located at the top of the image. Keratins are visualized as lines in the epithelial lining. Note that K8^−/−^ colon has only apical keratins and that K7 is expressed in the top-most cells of the crypts in K8^+/−^ and K8^−/−^. The overview is based on analysis of data presented in Fig. 2.
