# Supplementary material for: The Amount of Keratins Matters for Stress Protection of the Colonic Epithelium
Source: PLoS One. 2015 May 22;10(5):e0127436. doi: 10.1371/journal.pone.0127436 (PMC4441500; doi:10.1371/journal.pone.0127436)
Supplement: S3 Fig — (DOCX) [file pone.0127436.s003.docx]

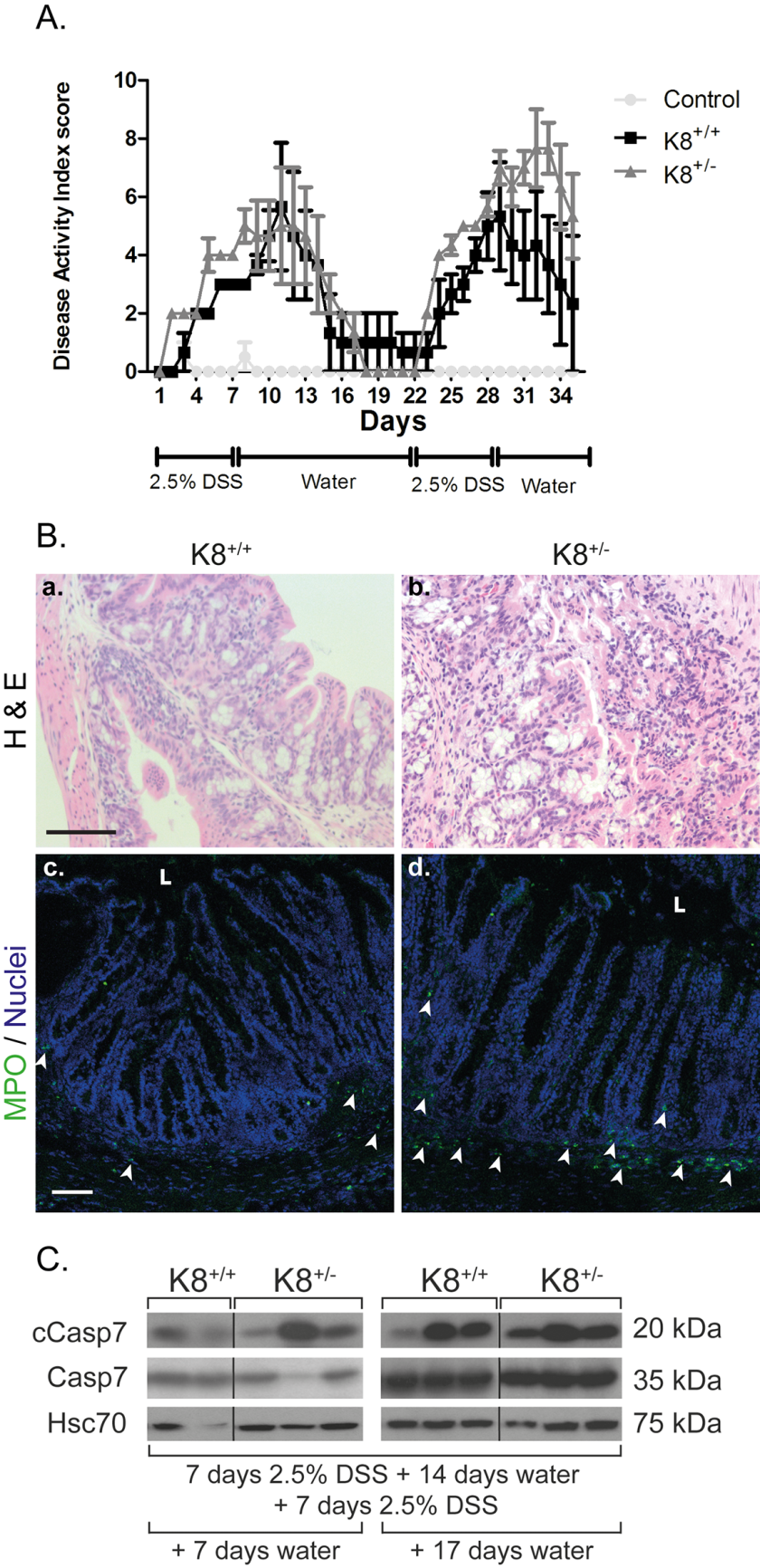


**Figure S3. K8^+/−^ mice are more sensitive to chronic 2.5% DSS treatment.** Mice were given two cycles of 2.5% DSS in drinking water followed by regular water as indicated in the figure. The K8^+/−^ and K8^+/+^ (A) disease activity score (DAI, see materials and methods), (B) histology (a-b) and MPO-staining (c-d) of representative mice shown in (A) on day 35 are shown. Scale bar in a and in c are 100 µm. Arrowheads in c-d indicate MPO (green) positive cells in the colonic epitheilum. Nuclei are stained blue. (C) The levels of cleaved caspase 7 (cCasp7), caspase 7 (Casp7) and Hsc70 in colon lysates at the end of the experiments are shown by western blotting.
