# Supplementary material for: The Amount of Keratins Matters for Stress Protection of the Colonic Epithelium
Source: PLoS One. 2015 May 22;10(5):e0127436. doi: 10.1371/journal.pone.0127436 (PMC4441500; doi:10.1371/journal.pone.0127436)
Supplement: S1 Table — (DOCX) [file pone.0127436.s004.docx]

**Asghar Table S1**

**Forward and reverse primers used for RT-PCR.**

| **Protein** | **Forward** | **Reverse** |
| --- | --- | --- |
| **β-Actin** | 5'-TGG CTC CTA GCA CCA TGA AGA-3' | 5'-GTG GAC AGT GAG GCC AGG AT- 3' |
| **K7** | 5'-GGA GAT GGC CAA CCA CAG-3' | 5'-GGC CTG GAG TGT CTC AAA CTT-3' |
| **K8** | 5'-TGA ATT TGT CCT CAT CAA GAA GG-3 | 5'-GGA TCT CCT CTT CAT GGA TCT G-3' |
| **K18** | 5'-AGA TGA CAC CAA CAT CAT CAC AAG G-3' | 5'-CTT CCA GAC CTT GGA CTTCCT-3' |
| **K19** | 5'-TGA CCT GGA GAT GCA GAT TG-3' | 5'-CCT CAG GGC AGT AAT TTCCTC-3' |
| **K20** | 5'-AGC TGA GAC GCA CCT ACC AG-3 | 5'-TGC GCT CCA GAG ACT CTT TC-3' |
| **K23** | 5'-TCA TGA AGA AAC GCC ATG AG-3' | 5'-CCT TGA AGT CAC TCG GCA AG-3' |
