# Supplementary material for: The Amount of Keratins Matters for Stress Protection of the Colonic Epithelium
Source: PLoS One. 2015 May 22;10(5):e0127436. doi: 10.1371/journal.pone.0127436 (PMC4441500; doi:10.1371/journal.pone.0127436)
Supplement: S2 Table — (DOCX) [file pone.0127436.s005.docx]

**Asghar Table S2**

**Colon length measurements in untreated and DSS-treated K8^+/+^ and K8^+/−^ mice.** Baseline colon lengths (in cm) for K8^+/+^, K8^+/−^ and K8^−/−^ mice, and colon lengths for K8^+/+^ and K8^+/−^ mice treated with an acute DSS-regimen (7 days 5% DSS + 2-4 days of water (measured at date of death), or chronic DSS (2.5% DSS + 14 days water + 2.5% DSS + 7 days water) are shown as averages ± SD. ***p< 0.001 when comparing 5% DSS-treated to untreated mice of the same genotype. No significant difference was seen between untreated mice of different genotypes, or between 5% DSS treated mice of different genotypes. *P<0.05 comparing K8^-/-^ baseline colon length to that of K8^+/+^. ND, not determined.

| **Genotype** | **Untreated mice,**  **colon length, cm** | **5% DSS-treated mice, colon length, cm** | **Chronic DSS-treated mice, colon length, cm** |
| --- | --- | --- | --- |
| **K8^+/+^** | 9.2 ± 1.4  (n=20) | 5.7 ± 1.2***  (n=7) | 7.7 ± 0.0  (n=2) |
| **K8^+/−^** | 8.6 ± 1.1  (n=12) | 5.0 ± 0.9***  (n=5) | 5.8 ± 0.8  (n=3) |
| **K8^−/−^** | 8.3 ± 0.9*  (n=14) | ND | ND |
